# Characterization of medical students recall of factual knowledge using learning objects and repeated testing in a novel e-learning system

**DOI:** 10.1186/s12909-014-0275-0

**Published:** 2015-01-24

**Authors:** Tiago Taveira-Gomes, Rui Prado-Costa, Milton Severo, Maria Amélia Ferreira

**Affiliations:** Department of Medical Education and Simulation, Faculty of Medicine of the University of Porto, Porto, Portugal; ALERT Life Sciences Computing, Vila Nova de Gaia, Portugal; Abel Salazar Biomedical Sciences Institute, University of Porto, Porto, Portugal

**Keywords:** Medical education, Memory retention, Computer-assisted instruction, E-learning, Tailored-learning, Spaced repetition, Test-enhanced learning, Judgment of learning, Curriculum evaluation, Blended-learning

## Abstract

**Background:**

Spaced-repetition and test-enhanced learning are two methodologies that boost knowledge retention. ALERT STUDENT is a platform that allows creation and distribution of Learning Objects named *flashcards*, and provides insight into student judgments-of-learning through a metric called ‘recall accuracy‘. This study aims to understand how the spaced-repetition and test-enhanced learning features provided by the platform affect recall accuracy, and to characterize the effect that students, *flashcards* and repetitions exert on this measurement.

**Methods:**

Three spaced laboratory sessions (s0, s1 and s2), were conducted with n=96 medical students. The intervention employed a study task, and a quiz task that consisted in mentally answering open-ended questions about each *flashcard* and grading recall accuracy. Students were randomized into study-quiz and quiz groups. On s0 both groups performed the quiz task. On s1 and s2, the study-quiz group performed the study task followed by the quiz task, whereas the quiz group only performed the quiz task. We measured differences in recall accuracy between groups/sessions, its variance components, and the G-coefficients for the *flashcard* component.

**Results:**

At s0 there were no differences in recall accuracy between groups. The experiment group achieved a significant increase in recall accuracy that was superior to the quiz group in s1 and s2. In the study-quiz group, increases in recall accuracy were mainly due to the session, followed by *flashcard* factors and student factors. In the quiz group, increases in recall accuracy were mainly accounted by *flashcard* factors, followed by student and session factors. The *flashcard* G-coefficient indicated an agreement on recall accuracy of 91% in the quiz group, and of 47% in the study-quiz group.

**Conclusions:**

Recall accuracy is an easily collectible measurement that increases the educational value of Learning Objects and open-ended questions. This metric seems to vary in a way consistent with knowledge retention, but further investigation is necessary to ascertain the nature of such relationship. Recall accuracy has educational implications to students and educators, and may contribute to deliver tailored learning experiences, assess the effectiveness of instruction, and facilitate research comparing blended-learning interventions.

**Electronic supplementary material:**

The online version of this article (doi:10.1186/s12909-014-0275-0) contains supplementary material, which is available to authorized users.

## Background

Medical education is a complex field where updates in medical knowledge, educational technology and teaching strategies intertwine in a progressive fashion [1-5]. Over the past decade there has been a shift in this field, where traditional instructor-centered teaching is yielding to a learner-centered model [6-9], in which the learner has greater control over the learning methodology and the role of a teacher becomes that of a facilitator of knowledge acquisition, replacing the role of an information provider [7,10-12].

Since the information learned by medical students is easily forgotten, it is important to design methodologies that enable longer periods of retention [13]. There is vast literature regarding the application of educational strategies [7,14-18], instructional design [11,19-22] and cognitive learning science [23-27] to the field of medical education in order to improve learning outcomes. Two promising approaches that emerge from that literature are ‘spaced repetition’ and ‘test-enhanced learning’.

### Spaced repetition

The term ‘spaced education’ describes educational interventions that are built in order to make use of the ‘spacing effect’ [13]. This effect refers to the finding that educational interventions that are distributed and repeated over time result in more efficient learning and retention compared to massed educational interventions [28-31]. Even though most of the evidence regarding the ‘spacing effect’ has been gathered in settings where interventions ranged from hours to days, there is some evidence suggesting that it can also generate significant improvements in longer-term retention [13].

Studies carried in the medical setting show that the application of such spaced interventions increase retention of learning materials. The interventions yielding these results have been designed as spaced-education games [32], delivery of content by email in spaced periods [13], blended approaches composed of face-to-face sessions and spaced contacts with on-line material [33], among others [23]. Cook et al. performed a meta analysis that regarded the application of spaced repetition and other methodologies on internet-based learning, and concluded that spaced repetition improves, at least, student satisfaction [11]. That work suggests that educators should consider incorporating repetition when designing internet-based learning interventions, even though the strength of such recommendations still needs reinforcement by further research [11].

### Test-enhanced learning

Even though tests are mainly used as a way to assess students, there is strong evidence that they stimulate learning by increasing retention of the information [34,35]. That has led Larsen et al. to define the term ‘test-enhanced learning’ to refer to interventions where tests are explicitly used to stimulate learning [36,37]. This approach is rooted in the observation that after an initial contact with the learning material, being tested on the material increases information retention more than reviewing that material again [37-39]. This effect increases with the number of tests [40] and the spacing of tests [41]. Moreover, tests composed of open ended questions (OEQs) have been shown to be superior to multiple choice questions (MCQs) for that purpose [42,43]. Providing the correct answer as feedback also increases the retention effect [44]. While most evidence indicates that immediate feedback is generally the most effective timing to maximize retention [45], there is recent evidence indicating that delayed feedback may have a stronger effect in some situations [46].

The test-enhancement effect is mostly explained by the recall effort required to answer the question, leading to superior retention [40]. In addition, there is also the indirect benefit of exercising judgments of learning (JOLs) that guide further study sessions [47]. JOLs, or meta-memory judgments, are made when knowledge is acquired or revisited [48]. Theories of self-regulated study claim that active learners use JOLs to decide whether to allocate further cognitive resources toward study of a given item or to move on to other items [49,50], thus supporting the indirect test-enhancement effect.

In the medical education setting, it has been shown that solving concrete clinical problems requires a strong grasp of the underlying factual knowledge that is inherent to the problem. Test-enhanced learning frameworks work particularly well for the retention of the factual knowledge required for higher order clinical reasoning [37,51]. It remains unclear, as in the case of spaced repetition, whether the test-enhancement effect can be maintained in the long term, as most of the evidence regards intervals ranging from weeks to months [40,46].

### Self-assessment and the ALERT STUDENT Platform

The creation of e-learning systems that enable systematic application of retention enhancement methodologies constitutes an important contribution to the information management axis of the core-competences for medical education [52] and may improve students ability to learn and retain the factual knowledge network required for effective clinical reasoning [27].

Based on the fact that there are few reports of systems implementing these principles in such a fashion [53], we have developed the platform ALERT STUDENT, a system that empowers medical students with a set of tools to systematically employ spaced repetition and test-enhanced methodologies to study learning materials designed in the form of Leaning Objects (LOs) [53]. This platform and the theoretical background supporting each of the features has been described in detail on a previous paper [53]. LOs are groupings of instructional materials structured to meet specific educational objectives [54] which are created using a set of guidelines to make content portable, interactive and reusable, [9,53-56] and have been shown to enhance learning [55].

The platform implements test-enhanced learning in the form of quizzes. These are composed of sets of OEQs about each of the LOs. The questions are meant to stimulate students to recall learned information, and therefore enable the measurement of JOLs. Typically, JOLs can be estimated as the prediction of the learner about how well it would recall an item after being presented the item [57]. Numerous methods exist to assess JOLs for different purposes [58]. The cue-only JOL, a method where the student must determine the recall of an item (in our case a LO) when only the cue (the OEQ) is presented at the time of judgment [58], is of particular interest to us. We extend this type of JOL to define a measurement named ‘recall accuracy‘. The recall accuracy is similar to the cue-only JOL because after being presented the cue and trying to retrieve the target, the student is presented the LO that contains the target. The student then grades the similarity between the retrieved target and the actual target. The process of measuring recall accuracy corresponds to the immediate feedback stage employed on test-enhanced learning approaches. This approach maximizes the potential of LOs and the OEQ to serve as learning material, recall cue and recall feedback.

To sum up, educators can use the platform to publish LOs, and students can apply the spaced repetition and test-enhanced methodologies on those LOs to hopefully improve their learning retention and direct study sessions effectively.

### Evaluation of education programs

Even though most educators value the importance of monitoring the impact of their educational interventions, systematic evaluation is not common practice, and is frequently based on inference measures such as extent of participation and satisfaction [59]. Additionally, most program evaluations reflect student cognitive, emotional and developmental experiences at a rather superficial level [59,60].

This issue also affects medical education [61]. Evaluation should drive both learning and curriculum development and demands serious attention at the earliest stages of change.

To make accurate evaluations of learning programs, it is essential to develop longitudinal databases that allow long term follow up of outcomes of interest [62]. In this line of thought we believe that recall accuracy information collected through the ALERT STUDENT platform in real-time may provide an additional resource to be included in student-oriented [61] and program-oriented [61] evaluation approaches, through the estimation of longitudinal student performance, and the determination of instruction and content fitness to student cohorts, respectively.

### Aims to this study

Since recall accuracy plays a key role in the learning method implemented by the ALERT STUDENT platform, this work aims, firstly, to characterize how recall accuracy evolves with usage of the spaced-repetition and test-enhanced learning tools in a controlled setting, and secondly, to characterize the extent to which students, LOs and intervention sessions contribute to the variation in recall accuracy. We hypothesize that recall accuracy improves along sessions, but we do not know how the contact with the system modulates it.

In addition we hypothesize that recall accuracy may constitute a relevant source of information to determine the learning difficulty of a LO for a given student cohort, and believe this information may contribute to the evaluation of the fitness of educational interventions. To elucidate this topic, we performed a G-Study to assess the agreement over the contribution of the LOs to recall accuracy scores, and performed a D-Study to characterize the conditions in which the number of students and repetitions of grading recall accuracy yield strong agreement on the difficulty of the LOs for the examined student cohort.

## Methods

The Faculty of Medicine of the University of *Porto* (FMUP) implements a 6-year graduate program. Applicants are mainly high school graduates. The first three years focus on basic sciences while the last three focus on clinical specialties. For the purpose of this work, content about the Golgi Complex was designed using lectures from the Cellular and Molecular Biology class, taught in the second semester of the first grade.

### ALERT STUDENT platform

The ALERT STUDENT the platform allows the creation and distribution of LOs named *flashcards*. These are self-contained information chunks with related OEQs. A *flashcard* is composed of a small number of information pieces and OEQs that correspond to one of the information pieces. Educators can put together ordered sequences of *flashcards* that describe broader learning objectives, thus forming high-order LOs denominated *notebooks*.

*Notebooks* are the units in which the spaced-repetition sessions and the test-enhanced learning tasks can be performed. Spaced-repetition tools are made available through a *study mode* feature that presents in order the complete set of *flashcards* belonging to a *notebook* in a study-friendly environment enriched with note taking, text highlighting, and a *flashcard* study priority cue based on personal recall accuracy from corresponding OEQs. The *flashcard* information and OEQs can be studied in this mode. Test-enhanced learning is achieved through the *quiz mode*, a complementary environment where retention of *flashcard* information can be self-assessed through recall accuracy using the OEQs as cues. Recall accuracy is graded for each question using a 4 point *likert* scale (0 - no recall, 1 - scarce recall, 2 - good recall, 3 - full recall). On every quiz session, the system picks one OEQ for every piece of information on every *flashcard*. OEQs are displayed one at a time. In case there is more than one OEQ for an information piece, the system picks one OEQ that has not yet been graded. When all the OEQs have been graded for a given information piece, the system picks the OEQ with the lowest recall accuracy. At the end of a *quiz mode* session, the student is presented the set of *flashcards* and OEQs for which recall accuracy was 0.

### Pilot study

A pilot study was performed to design a *notebook* that could be studied in 20 minutes. 5th grade students (n = 6) were assigned to a read a *notebook* with 30 *flashcards* created using lecture material about the Golgi Complex. The final *notebook* was created using the *flashcards* that the students were able to study within the time limit. That *notebook* consisted of the first 27 *flashcards*, totaling 37 information pieces and 63 OEQs. Each *flashcard* contained one or two pieces of information, sometimes accompanied by an image - there were 5 images in total. Each piece of information in a *flashcard* corresponded to a set of 1 to 4 OEQs. This *notebook* script is available as a Additional file 1 to this paper.

Furthermore, in order to estimate the sample size, 2nd grade students (n = 2), 4th grade (n = 2), and 5th grade (n = 2) medical students were asked to grade their recall accuracy for the 63 OEQs. The 4th and 5th year students knowledge was assumed to correspond to a low recall accuracy about the Golgi, and was expected to represent the mean recall accuracy of a similar student sample before the research intervention. 2nd grade medical students knowledge was assumed to correspond to a high recall accuracy about the Golgi, and was expected to represent the mean the recall accuracy of a student sample after the research intervention.

The average percentage difference in recall accuracy between the two student groups was 41%. Finding a similar difference in mean recall accuracy before and after an intervention using the study and quiz tools was assumed to be a reasonable expectation. Thus, the sample size required to discriminate statistical significance under such circumstances was n = 48, assuming a power of 80% and a significance level of 0.05. The sample size was incremented to n = 96 to take advantage of the laboratory capacity.

### Intervention design

Ninety-six (n = 96) students from the 4th and 5th grades of our school were randomly picked from the universe of enrolled students (approx. 500), and were contacted via email to participate one month prior to this study. Two students promptly declined to participate and two more students were randomly picked. Students were assigned into ‘study-quiz’ group or ‘quiz’ group using simple randomization.

The intervention employed a study task and a quiz task. The study task consisted in studying the Golgi *notebook* during 20 minutes using the *study mode*. The students were able to take notes and highlight the text. The quiz task consisted in using the *quiz mode* to answer the OEQs about the Golgi and grade recall accuracy, within 15 minutes. Before each task students were instructed on the purpose of each task and the researcher exemplified each of the tasks in the system. Students performed each task alone. Doubts raised by the students concerning platform usage were cleared by the researcher.

Three laboratory sessions (s0, s1 and s2) of 1 hour duration were carried with one week intervals. On s0, both groups performed the quiz task. On s1 and s2, the quiz group performed the quiz task alone, and the study-quiz group performed the study task immediately followed by the quiz task. Since the platform implements a study workflow centered on performing the study task followed by the quiz task, the study-quiz group was created to indirectly measure changes in recall accuracy attributable to the study task. The quiz group describes the changes in recall accuracy that are attributable to the quiz task. This procedure is detailed in Table 1.

**Table 1:** **Study design**

**Session**	**Quiz group (n = 49)**	**Study-quiz group (n = 49)**
0	Quiz - 15 min	Quiz - 15 min
1 week interval		
1	Quiz - 15 min	Study - 20 min
		Quiz - 15 min
1 week interval		
2	Quiz - 15 min	Study - 20 min
		Quiz - 15 min

Representation of the study intervention. Participants (n = 96) were split into quiz and study-quiz groups by simple randomization. During s0 both groups performed the quiz task during 15 minutes. On s1 and s2 the quiz group performed the quiz task again for 15 minutes. The study-quiz group performed a 20 minute study task, immediately followed by the 15 minute quiz task. Sessions were separated by one week intervals.

### Sample characterization

In session s0 both groups filled a survey to characterize the student sample. Measured factors were gender, course year, preferred study resource for Cellular Biology, computer usage habits, Cellular Biology grade, mean course grade, and average study session duration during the semester and during the exam season. The Cellular Biology grade was assumed to be the grade that best estimated prior knowledge about the Golgi. These factors were added to characterize the study sample and assess eventual dissimilarities in the sampling of the two groups.

### Statistical Analysis

For each session and group, *flashcard* recall accuracy was computed as the mean recall accuracy of the OEQs belonging to a *flashcard*.

In order to characterize the changes in recall accuracy across sessions, we used univariate repeated-measures analysis of variance (ANOVA). Groups were used as between-subjects factor. Session and *flashcard* were used as within subject factor. Repeated contrast (s0 vs s1 and s1 vs s2) was used to evaluate the sessions and the session interaction effect.

In order to estimate the variance components for the recall accuracy for both groups, a random effects model was used and the *flashcard*, the session and the student were used as random variables. The estimation was performed using the Restricted Maximum Likelihood method. In order to estimate the agreement on the *flashcard* component its specific G-coefficient was calculated. A D-Study was performed to characterize the agreement on the *flashcard* component for different student and session counts. Guidelines for interpreting G-coefficients suggest that values for relative variance between 81 - 100% indicate almost perfect agreement, 61 - 80% substantial agreement, 41 - 60% moderate agreement, 21 - 40% fair agreement, and values less than 21% depict poor or slight agreement [63].

The statistical analysis was performed using R software. The package ‘lme’ was used to compute the random effects model.

This study was approved by the Faculty of Medicine University of *Porto*/*São João* Hospital Ethics Committee in compliance with the Helsinki Declaration. Collected data was analyzed in an anonymous fashion. It was not possible for the researchers to identify the students during any phase of the data analysis.

## Results

### Study sample characterization

94 participants completed the session s0. 1 participant in the study-quiz group and 1 participant in the quiz group did not complete session s1 and were excluded from the study. By the end of the study there were 47 participants in each group.

59 participants were female and 35 participants were male. 44 participants were enrolled in the 4th grade and 53 were enrolled on the 5th grade. The preferred study resources for Cellular Biology were *Professor texts* (n = 36), followed by *Lecture notes* (n = 24), *Lecture slides* (n = 23) and finally the *Textbook* (n = 11). Most participants reported using computers every day (n = 78). Average course grade was 68%, and the average Cellular Biology grade was 64% - equivalent results for the student population were 65% and 62% respectively, representing a fair score. Participants reported daily study sessions during the semester to last on average 3.0 hours and daily exam preparation study sessions to last on average 9.5 hours. No significant differences between the study-quiz and quiz groups were found for any of the sample characterization factors. These results are described in further detail in Table 2.

**Table 2:** **Study sample characterization**

	**Total**	**Control**	**Experiment**	**p**
Gender	n (%)	n (%)	n (%)	
Female	59 (62.8)	28 (59.6)	31 (65.9)	0.670
Male	35 (37.2)	19 (40.4)	16 (34.1)	
Course year	n (%)	n (%)	n (%)	
4th year	44 (46.8)	23 (48.9)	21 (44.7)	0.836
5th year	50 (53.2)	24 (51.1)	26 (55.3)	
Preferred resource	n (%)	n (%)	n (%)	
Professor texts	36 (38.3)	17 (36.2)	19 (40.4)	0.898
Lecture notes	24 (25.5)	12 (25.5)	12 (25.5)	
Lecture slides	23 (24.5)	13 (27.7)	10 (21.3)	
Textbook	11 (11.7)	5 (11.6)	6 (12.8)	
Computer usage	n (%)	n (%)	n (%)	
Everyday	73 (77.7)	37 (78.2)	36 (76.6)	0.193
Not everyday	21 (22.3)	10 (21.2)	11 (23.4)	
Grades	Mean (SD)	Mean (SD)	Mean (SD)	
Cellular biology	64 (6)	65 (8)	64 (8)	0.102
Course average	68 (5.5)	69 (5.5)	68 (5.5)	0.433
Daily study hours	Median (IR)	Median (IR)	Median (IR)	
During semester	3.0 (2.5)	3.0 (2.0)	3.0 (2.0)	0.628
During exam season	9.5 (2.0)	10.0 (2.0)	8.0 (2.0)	0.307

Cellular Biology Grade and Course Average are displayed in a 0-100% grading scale. SD - Standard Deviation; IR - Interquartile range.

### Recall accuracy characterization

Mean recall accuracy increased from 25% in s0, to 53% in s1, to 62% in s2. In the quiz group, mean recall accuracy increased from 24% in s0 to 33% in s1 (p <0.001) to 42% in s2 (p <0.001). In the study-quiz group, recall accuracy increased from 27% at s0 to 73% at s1 (p <0.001) to 82% at s2 (p <0.001). At session s0, there were no differences in recall accuracy between groups. During s1 and s2, recall accuracy differences between groups were statistically significant (p <0.001). The study-quiz group achieved a sharper increase in recall accuracy than the quiz group. The increase in recall accuracy was greater between s0 and s1 for both groups. In respect to the study-quiz group, recall accuracy had a relative increase of 63% from s0 to s1. Between s1 and s2 there was a relative increase of 12% in recall accuracy for that group. The quiz group had a relative increase of 27% between s0 and s1, and a relative increase of 21% from s1 to s2. These results are described in further detail in Table 3.

**Table 3:** **Recall accuracy per session and group**

	**Total (%)**	**Control (%)**	**Experiment (%)**	
	Mean (SD)	Mean (SD)	Mean (SD)	p^1^
s0	25.3 (18.7)	24.0 (16.7)	27.0 (17.7)	0.924
s1	53.0 (22.3)	33.0 (18.0)	72.7 (18.3)	<0.001
s2	62.3 (21.7)	42.0 (20.7)	82.3 (15.0)	<0.001
p^2^	<0.001	<0.001	<0.001	< 0.001^3^

SD - Standard Deviation; ^1^Differences in recall accuracy between study-quiz and quiz group; ^2^Differences in recall accuracy between pairwise sessions; ^3^Interaction effect between session and group.

Regarding the ANOVA, the session and group Dfs equaled 1, Sum square/Mean square difference values were 56.5 for the session, and 23.5 for the group. F-values were 292.2 for the session and 121.2 for the group. Eta-squared values were 0.32 for the session and 0.27 for the group.

Regarding the components of variance for recall accuracy in the quiz group, the largest one was the *flashcard* (34.7%). The participant and session components explained a small proportion of variance (15.1% and 8.2%, respectively) reflecting small systematic differences among participants and sessions. The residual component accounted for 41.2% of the total variance. These results are described in further detail in Table 4.

**Table 4:** **Components of variance of recall accuracy for the quiz group**

**Component**	**n**	**Variance**	**SD**	**%** ^**1**^
Participant	47	0.165	0.406	15.1%
*Flashcard*	27	0.377	0.614	34.7%
Sessions	3	0.089	0.299	8.2%
Residual	3440	0.456	0.676	41.2%

SD - Standard Deviation; ^1^Percentage of total variance.

In respect to the components of variance for recall accuracy in the study-quiz group, the most prominent factor was the session (49.6%). The participant and *flashcard* components explained a small proportion of variance (5.1% and 15.3%, respectively). The residual component accounted for 30.0% of the variance. These results are described in further detail in Table 5.

**Table 5:** **Components of variance of recall accuracy for the study-quiz Group**

**Component**	**n**	**Variance**	**SD**	**%** ^**1**^
Participant	47	0.083	0.288	5.1%
*Flashcard*	27	0.249	0.499	15.3%
Sessions	3	0.812	0.900	49.6%
Residual	3422	0.493	0.702	30.0%

SD - Standard Deviation; ^1^Percentage of total variance.

For both groups two-way and three-way interactions were computed and explained a very small fraction of total variance.

G-coefficient for the *flashcard* variance component was 91% in the quiz group, indicating almost perfect agreement. Regarding the study-quiz group, the coefficient value was 47%, indicating moderate agreement.

**Figure 1:**
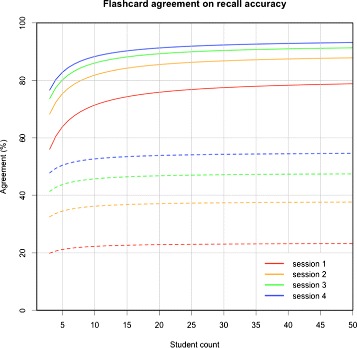
**D-Study for the agreement on the** ***Flashcard*** **variance component of recall accuracy.** G-coefficient for the *flashcard* component of recall accuracy, using different combinations of number of students (x axis) and sessions (colored curve sets). The stroked curve set represents quiz group agreement, and the dashed curve set represents study-quiz group agreement. It can be seen that with a small number of students and sessions of using the study and quiz modes (dotted curve set) or quiz mode alone (stroked curve set), substantial (>60%) and strong (>80%) *flashcard* agreements on recall accuracy can be obtained, respectively. High *flashcard* agreement for recall accuracy denotes that systematic differences in *flashcards* explain recall accuracy differences. This information may be useful to inform educators of learning materials that may require review.

The D-Study performed for the *flashcard* variance component showed that almost perfect agreement (>80%) can be achieved by having 10 students perform the quiz task on 2 spaced sessions. Circumstances to obtain such levels of *flashcard* agreement for the study and quiz task would require unfeasible numbers of students and sessions. Figure 1 plots the D-Study agreement curves for the *flashcard* variance component in both study-quiz task and quiz task alone, for different student and session counts.

## Discussion

It was unclear what difference to expect in terms of recall accuracy between groups and between sessions. We selected a basic science topic and 4th and 5th grade medical students, in order to maximize the odds of a low degree of prior knowledge. We chose the Golgi Complex because the majority of the curriculum does not build directly on this concept, and thus it was likely a forgotten topic. This was important because the lowest the a prior knowledge before our intervention, the smaller student sample would be required to discriminate significant differences in recall accuracy during the study sessions, thus rendering this study feasible.

### Evolution of recall accuracy across sessions

There is an effect on recall accuracy reported by students along sessions. It was expected that the study-quiz group would out-perform the quiz group in terms of recall accuracy, at least on s1. Since the quiz task provides the learning materials as the correct answers to the OEQs and additional feedback at the end of the task, it has high learning value. Because we used a 4 point scale to grade recall accuracy, it was reasonable to consider the hypothesis that the quiz task provides enough learning value to master the content and thus expect both groups to report similar recall accuracy results.

The recall accuracy increase was stronger in session s1 for the study-quiz group. It was expected to see an increase in this session since the content was tailored to be fully covered within the 20 minute time limit. The strong gain indicates that this session was the one that accounted for the greatest increase in recall accuracy.

Findings by Karpicke et al. suggest that the testing effect plays an essential role in memory retention, and that after an initial contact with the learning material it is more beneficial to test rather than re-study the material [40]. In addition, since using open-ended assessment questions as a means to learn improves knowledge retention [37,39,47], it was unclear how strong would that increase be in the quiz group. However that increase was only a modest one. That finding might be explained, at least in part, by minimization of the cueing effect - the ability to answer questions correctly because of the presence of certain questions elements [64,65] - through the usage of different questions for each information piece. OEQs are known to minimize cueing [65,66] and in addition, the different questions, although having the same content as answer, minimized that effect. This shows that pairing OEQs with LOs increases the value of the learning material.

In our study we found that recall accuracy increased more in the study-quiz than in the quiz group. If we assume that recall accuracy represents knowledge, then the most likely explanation for higher the increase in recall for the study-quiz group is the additional time-on-task. We were concerned that, because the metric is a subjective one, repeated contact with the content would cause the recall accuracy value to overshoot to nearly 100% after the first contact, regardless of prior knowledge or the time-on-task. However, recall accuracy evolved along sessions according to the underlying variables: recall accuracy at s0 was low because the student cohort did not have any formal contact with the Golgi over 2 years; the study-quiz group - with longer time-on-task - had higher results than the quiz group; recall accuracy improved along the sessions for both groups in part because of the effect of previous sessions.

Thus recall accuracy evolved in accordance to the factors influencing learning.

### Adequacy of recall accuracy as a measurement of knowledge

The consistent differences in recall accuracy between groups give and indication that this measurement, although being of subjective nature, seems to be positively related with knowledge acquisition.

Karpicke et al. has shown that in a controlled setting, students cannot reliably predict how well they will perform on a test based on their JOL [40]. Other studies conducted in ecological settings also have shown that the relationship of knowledge self-assessment with motivation and satisfaction are stronger than with cognitive learning [67-69]. Additional research found that in a blocked practice situation learners tend to be overconfident and JOLs are often unreliable [70].

Our study design differed from the classical designs for studying the effects of spaced repetition, knowledge retention and JOLs [28] because it was intended to describe recall accuracy evolution in a use-case similar to the real-world use of the system. Therefore, available evidence may not be completely applicable to this study. However, based on our results, we cannot completely refute the hypothesis that recall accuracy is independent of knowledge acquisition and dependent on affective factors. It is possible, though unlikely, that affective factors introduce a systematic error in recall accuracy grading. The colorful nature and intensity of such factors would most likely lead to a random error rather than systematic variation. This finds support in our results regarding recall accuracy variance components, since the *flashcard* component contributed substantially more than the participant component to the total variance. In addition, it is well known that higher time-on-task is one of the most important determinants of learning [71]. Because recall accuracy was higher on the study-quiz group - with greater time-on-task - this is likely mainly explained by the learning effect.

Furthermore, other studies have measured JOLs differently than in this study. While other approaches typically measure JOL by requiring the subject to predict how well would they perform when tested in the future [29,40,70], our approach focuses on requiring subjects to compare their answer with the *flashcard* containing the correct information. Because our approach does not require a future projection and is additionally performed in the presence of both the recalled and correct answers, it is unlikely to vary independently of the learning effect.

Thus, we hypothesize that measuring recall accuracy immediately after the recall effort and in the presence of the correct answer may help students make sound JOLs. However further work is needed to compare recall accuracy with an objective measurement of knowledge, such as a MCQ test, in order to prove that hypothesis. Assuming a relationship between both variables is found, it would also be relevant to understand how different degrees of recall accuracy map to different degrees of knowledge.

### Recall accuracy components of variance

Regarding the quiz group, the recall variance was mainly affected by the differences in *flashcard* and by the differences in participants. This indicates, firstly, that systematic differences in the *flashcards* were mainly responsible for the variation in recall scores, and secondly, to a smaller extent, differences between participants, possibly regarding affective and knowledge factors also played a role. The effect of the multiple sessions accounted little for the increase in recall accuracy over the sessions. The high G-coefficient for the *flashcard* variance component indicates the *flashcards* are very well characterized in terms of recall accuracy under these circumstances. Thus, factors intrinsic to the content, such as its size, complexity, or presentation, are very likely responsible for differences in recall accuracy between *flashcards*.

Assuming the recall accuracy is related to knowledge acquisition, systematic differences in recall accuracy between *flashcards* can indicate which materials are harder to learn and which materials are easy. Using this information to conduct revisions of the learning material may be useful to find content that would benefit from redesign, adaptation, or introductory information.

With respect to the study-quiz group, the contact with the content over multiple sessions was the main driver of recall accuracy improvement. Participant features had little effect in the increase recall accuracy over sessions and the *flashcard* features also accounted for less effect than in the quiz group. This suggests that the students in the study-quiz group increased their knowledge about the content and their prior knowledge had little effect in the learning process when using the study tools. This effect is most likely explained by the additional time-on-task of the study-quiz group. In addition, some of the effect may also be explained by findings in other studies that show that there is benefit in using repeated testing with study session in order to enhance learning [37,39,47].

### Potential implications to educators

The way in which content can be organized to optimize learning has been extensively studied [26,52,54,72-74]. This study demonstrates how LOs can be of value for both study and self-assessment when combined with OEQs. The detailed insight on recall accuracy can be used by educators to classify LO difficulty and estimate the effort of a course. By providing a diagnostic test on the beginning a course in the form of the quiz task, educators can get a detailed snapshot of the material difficulty for the class. This data can be useful to evaluate educational interventions at a deeper level [62]. Because the platform can be used by the students to guide learning on their own, educators can access real-time information of recall accuracy and use it to tailor the structure of the class to better meet the course goals. Furthermore, research has identified the delivery of tailored learning experiences as one of the aims that blended education approaches have yet fully reached [75].

In a hypothetical scenario where students repeatedly study and quiz, it is expected that the main component of recall accuracy variance is the session count. Deviation from such a pattern could suggest flaws in content design, excessive course difficulty or other inefficacies in teaching and learning methodologies. Sustained increases in recall accuracy mainly explained by the session would inform the educator of a continuous and successful commitment of the students. If educators take constructive action from such observations then a positive feedback cycle between student engagement and the success of the learning activity would be established. Because students know educators can take real-time action based on their progress, they engage more strongly in the learning activities. Stronger engagement will lead to better learning outcomes, that will lead to further tailored action by the teacher. Indeed, student engagement is the main driver of learning outcomes [76]. Providing tools that can foster such engagement is key to achieve successful learning [77,78].

### Potential implications to learners

Students need tools to help retain knowledge for longer periods and easily identify materials that are more difficult to learn [13]. This goal may be achieved by providing learners with personal insight on their learning effectiveness, using personal and peer progress data based on self-assessment results [55].

The past recall accuracy can be used as an explicit cue to guide the learning process and help managing study time. Since JOL measurements are implicitly used by learner to guide the learning task [29,41], an explicit recall accuracy cue displayed for each *flashcard* in the form of a color code can improve the value of the JOL [53]. The feedback that is thus formed between the quiz and the study task further promotes the spaced repetition of study and self assessment sessions and can improve student engagement, the main driver of successful learning. This is even more important at a time where students need to define tangible goals that allow them cope with course demands [79].

Each *flashcard* holds the recall accuracy for each student for each assessment. Increasing spaced repetitions of study and quiz increase the available recall accuracy data. Since *notebooks* can be constructed using any available *flashcard*, it is possible to create *notebooks* that include *flashcards* for which recall accuracy is already available. Therefore, advanced *notebooks* requiring background knowledge can include an introductory section composed of the most relevant *flashcards* about the background topics. This implies that without previous contact with the advanced *notebooks*, an estimate of how well the student recalls the background topics is already available. This increases the value of learning materials by fostering reutilization and distribution of LOs between different courses, educators and students [53-55,80] and promoting educator and student engagement [77].

### Proposal for curricular integration

In recent years multiple educational interventions have described the benefits of implementing blended learning methodologies in medical education, namely in radiology [81], physiology [18], anatomy [17] and others [82,83]. However, the design of these interventions varies widely in configuration, instructional method and presentation [75]. Cook asserted that little has been done regarding Friedman’s proposal [84] of comparing computer based approaches rather than comparing against traditional approaches [75].

The platform ALERT STUDENT intends to add value to the blended learning approach, through the collection of recall accuracy data, and prescription of a method that can be systematically applied in most areas of medical knowledge. Over this platform, interventions with different configuration, instructional method or presentation can be developed, and thus allow sound comparison between computer assisted interventions and comparison between different fields of medical knowledge. The platform does not intend, however, demote the usage of other tools, rather it intends to potentiate their usage. As an example, the platform could be used to deliver the learning materials and provide the study and quiz features, that would act in concert with MCQ progress tests during class. Educators could use information about recall accuracy and number of study and quiz repetitions to gain insight on the relationship between test results and student effort. That information would be relevant to help educators mentor students more effectively. Again, the information brought by recall accuracy could be helpful to tailor other instructional methods and thus drive student satisfaction and motivation.

### Limitations and further work

This work has several limitations. Recall accuracy cannot be granted to correspond to knowledge retention. As previously mentioned, additional research is required to investigate the relationship between the two. In the light of our findings, it also becomes relevant to characterize recall accuracy in ecological scenarios and multiple areas of medical curriculum, under larger learning workloads.

We have indirectly characterized the effect of the study task on the recall accuracy. We expect however that an equivalent time on the quiz task alone would yield higher effects in recall accuracy, in consonance with the findings by Larsen et al. [36,37]. That is also a matter that justifies further investigation.

The system works around factual knowledge, therefore it is only useful in settings that require acquisition of such knowledge. Complex competences such as multi level reasoning and transfer cannot be translated in terms of recall accuracy. Ways in which the system could be empowered to measure such skills would constitute important improvements of the platform.

## Conclusions

The present study focus on measuring recall accuracy of LOs using OEQs in a laboratory setting through the ALERT STUDENT platform. We found that the quiz task alone led to a modest increase on recall accuracy, and that the study-quiz task had high impact in recall accuracy. The session effect was the main determinant of recall accuracy on the study-quiz group, and the *flashcard* and participant effects determined most of the increase in recall accuracy in the quiz group. We concluded that recall accuracy seems to be linked with knowledge retention and proposed further investigation to ascertain the nature of this relationship. Recall accuracy is an easily collectible measurement that increases the educational value of LOs and OEQs. In addition, we have discussed the educational implications of providing real-time recall accuracy information to students and educators, and proposed scenarios in which such information could be useful to deliver tailored learning experiences, assess the effectiveness of instruction, and facilitate research comparing blended learning interventions.

The present findings will be explored in more detail in future work, as they may help future physicians and medical schools meet the challenge of information management [52] and instilling a culture of continuous learning, underpinning the core competencies outlined for XXI century physicians [3,4].

## Additional file

Additional file 1
**The Notebook script.**

## Abbreviations

ANOVA: Analysis of variance
FMUP: Faculty of Medicine University of *Porto*
s0: Study session 0
s1: Study session 1
s2: Study session 2
JOL: Judgment of learning
MCQ: Multiple choice question
OEQ: Open ended question
