# Supplementary material for: Characterization of medical students recall of factual knowledge using learning objects and repeated testing in a novel e-learning system
Source: BMC Med Educ. 2015 Jan 24;15:4. doi: 10.1186/s12909-014-0275-0 (PMC4326410; doi:10.1186/s12909-014-0275-0)
Supplement: Additional file 1 — The Notebook script. [file 12909_2014_275_MOESM1_ESM.pdf]

**Topic**	**1**	**The Golgi network**
**Flashcard**	**1**	
Piece	1	The Golgi network is involved in protein processing, trafficking and the synthesis of glycolipids and polysaccharides.
Question	1	*In what processes is the Golgi complex involved?*
Piece	2	The proteins are transported from the endoplasmic reticulum (ER) to the cis-Golgi network and complete the process of maturation in the trans-Golgi network, where the proteins are packed into vesicles to be transported to the lysosomes (via endosomes), the plasmatic membrane or to the cell exterior.
Question	2	*Where are the proteins from the ER transported to?*
Question	3	*Where do the proteins from the ER come from?*
Question	4	*Where do the proteins complete their maturation process?*
Question	5	*Where are the proteins that pass through the ER sent to?*
**Flashcard**	**2**	
Piece	3	The designation "Golgi Apparatus" is used to refer all the Golgi networks in the same cell.
Question	6	*What does the designation “Golgi Apparatus” refer to?*
**Flashcard**	**3**	
Piece	4	Protein maturation by n-glycosylation occurs during the transport along the Golgi network.
Question	7	*Through which process does the maturation in the Golgi network occur?*
Question	8	*When does the protein n-glycosylation takes place?*
**Flashcard**	**4**	
Piece	5	The Golgi network synthesizes glycolipids, sphingomyelin and complex polysaccharides that make part of the plant cell wall.
Question	9	*What are the substances synthesized in the Golgi network?*
**Flashcard**	**5**	
Piece	6	The Golgi network is composed by a group of cisterns (dictyosomes) and vesicles.
Question	10	*What is the Golgi network composed of?*
**Topic**	**1.1**	**Golgi network compartments**
**Flashcard**	**6**	
Piece	7	There are three types of functionally distinct compartments in the Golgi network:<br>cis-Golgi face<br>cisterns (subdivided into cis, medial and trans)<br>trans-Golgi face
Question	11	*Which compartment types make up Golgi network?*
Piece	8	The vesicles from the ER fuse, forming an intermediate compartment between the RE and the Golgi, the ERGIC, that transports proteins to the cis-Golgi network.
Question	12	*What is the name of the intermediate compartment between the ER and the Golgi network?*
Question	13	*Where are the proteins from the EREGIC transported to?*
Image	1	The Golgi network compartments

**Flashcard**	**7**	
Piece	9	The cis, medial and trans cisterns are the sites where the majority of the processing reactions occur.
Question	14	*Which are the cisterns where the majority of the processing reactions occur?*
Piece	10	The trans-Golgi network works as a center for triage and distribution of the proteins to the endosomes, the lysosomes, the plasmatic membrane or the exterior of the cell.
Question	15	*What is the specific of the trans-Golgi network?*
**Flashcard**	**8**	
Piece	11	Proteins from the ER enter through the cis face, also known as formation face. This face is convex and oriented towards the cell nucleus.
Question	16	*Where is the point of entrance on the Golgi network for proteins coming from the ER?*
Question	17	*What are the characteristics of the cis-Golgi face?*
Piece	12	The proteins that are transported along the Golgi network, exit through the concave trans-Golgi face, also designated maturation face. These proteins are sent to endosomes, lysosomes, the plasmatic membrane and the exterior of the cell, as illustrated in the picture.
Question	18	*From which point do carried proteins leave the Golgi network?*
Question	19	*What are the characteristics of the trans-Golgi face?*
Question	20	*What are the destination locations of the proteins that leave the Golgi network?*
Image	2	Electron microscopy of the Golgi network
**Topic**	**1.2**	**Transport from the endoplasmic reticulum to the Golgi complex**
**Flashcard**	**9**	
Piece	13	The proteins that belong to the ER are named resident proteins. These proteins are transported in a non-specific manner from the ER to the Golgi, and are recovered via retrograde transport to the ER.
Question	21	*How are resident proteins from the ER recovered from the Golgi network?*
Piece	14	Resident proteins from the ER are identified by a retention signal on its C-terminus that signals them to retrograde transport
Question	22	*What is the signal that identifies ER resident proteins?*
Question	23	*Where is the signal that identifies a protein as part of the ER located?*
Image	3	Traffic between the ER and the Golgi network
**Flashcard**	**10**	
Piece	15	The soluble ER resident proteins retention signal consists of 4 amino acids in KDEL sequence (Lys-Asp-Glu-Leu).
Question	24	*What is the amino acid sequence of the retention signal of soluble ER resident proteins?*
Piece	16	The KDEL sequence links specifically to the KDEL receptor, on the ERGIC or Golgi, which allows resident protein packaging in COPI coated vesicles for retrograde transport to the ER.
Question	25	*To which receptor does the retention signal of the soluble proteins links to?*
Question	26	*Where does the retention signal binding to the soluble protein receptor occurs?*
Question	27	*In which vesicle type ER resident proteins are transported back to the ER?*

**Flashcard**	**11**	
Piece	17	Transmembrane proteins retention signal consists of 2 lysine residues followed by other 2 other amino acids (KKXX). It links directly to COPI coated vesicles that allow the retrograde transport to the RE.
Question	28	*What is the amino acid sequence of the transmembrane resident proteins?*
Question	29	*What is the type of vesicles that transmembrane resident proteins link to?*
**Flashcard**	**12**	
Piece	18	Proteins and lipids coming to the Golgi-network from the ER are first transported to the ERGIC and then to the cis-Golgi network via COPI coated vesicles.
Question	30	*Which are the structures in which proteins and lipids are passed to from the ER to the Golgi network?*
**Topic**	**1.3**	**Metabolism of lipids and polysaccharides**
**Flashcard**	**13**	
Piece	19	In addition to glycoprotein processing, the Golgi network is also involved in the lipidic metabolism and in particular the synthesis of glycolipids and sphingomyelin.
Question	31	*What other process is the Golgi network involved in addition to glycoprotein processing?*
**Flashcard**	**14**	
Piece	20	Sphingomyelin results from the addition of a phosphorylcholine group to a ceramide molecule.
Question	32	*What is the residue that produces sphingomyelin when added to ceramide?*
Question	33	*What is the residue that produces sphingomyelin when added phosphorylcholine group?*
Question	34	*Which molecules compose sphingomyelin?*
**Flashcard**	**15**	
Piece	21	Glycoproteins result from the addition of carbohydrates to ceramide.
Question	35	*How are glycolipids formed?*
Question	36	*What is the residue that produces glycolipids when added carbohydrates?*
**Flashcard**	**16**	
Piece	22	In plants, the Golgi network is mainly involved in the synthesis of polysaccharides that form the nuclear wall.
Question	37	*In which process is the Golgi network mostly involved in plants?*
**Topic**	**2**	**Maturation of proteins by O-linked glycosylation**
**Flashcard**	**17**	
Piece	23	Another aspect of the processing of glycoproteins in the Golgi network consists of the addition of carbohydrates to the OH group on the serine and threonine residues present in specific peptidic sequences (O-linked glycosylation).
Question	38	*What does the O-linked glycosylation process consists of?*
**Flashcard**	**18**	
Piece	24	The O-linked glycosylation process is catalyzed by a series of glycosyltransferases that add firstly a n-acetylgalactosamine residue and after a variable number of carbohydrates, usually up to 10 residues.
Question	39	*What are the proteins involved in the O-linked glycosylation process?*
Question	40	*What is the first residue added by the enzymes that catalyze the O-linked glycosylation process?*
Piece	25	In some cases these residues are further modified by the addition of sulphate groups.
Question	41	*What residues can be further added to the carbohydrates of the O-linked glycosylation matured proteins?*

**Flashcard**	**19**	
Piece	26	Some cytosolic and nuclear proteins are processed by O-linked glycosylation.
Question	42	*What are the final locations of the proteins processed by O-linked glycosylation?*
**Topic**	**3**	**Maturation of proteins by n-linked glycosylation**
**Flashcard**	**20**	
Piece	27	One of the most important processes in the maturation of the glycoproteins in the Golgi network consists of the modification of the n-linked oligosaccharides added in the ER by an ordered sequence of reactions in each cistern. In the proteins destined to the plasmatic membrane or secretion, the first modification occurs via removal of 3 residues of mannose in the cis-Golgi network.
Question	43	*What is the first modification that occurs in the proteins destined to the plasmatic membrane or secretion?*
Question	44	*Where does the first modification occur in the proteins destined to the plasmatic membrane or secretion?*
**Flashcard**	**21**	
Piece	28	In the proteins destined to the plasmatic membrane, the second step occurs in the medial-Golgi network and consists of the removal of 2 residues of mannose and the addition of 3 residues of n-acetylglucosamine and fucose.
Question	45	*What is the second modification that occurs in the proteins destined to the plasmatic membrane or secretion?*
Question	46	*Where does the second modification occur in the proteins destined to the plasmatic membrane or secretion?*
**Flashcard**	**22**	
Piece	29	In the proteins destined to the plasmatic membrane, the last step takes place in the trans-Golgi network, and consists of the addition of 3 residues of galactose and the addition of n-acetylneuraminic acid to each galactose residue.
Question	47	*What is the last modification that occurs in the proteins destined to the plasmatic membrane or secretion?*
Question	48	*Where does the last modification occur in the proteins destined to the plasmatic membrane or secretion?*
Image	4	Processing of n-linked oligosaccharides in the Golgi complex cisterns
**Flashcard**	**23**	
Piece	30	The degree of processing of the n-linked oligosaccharides depends on:<br>The structure of the proteins in the Golgi network<br>The quantity of enzymes in the Golgi network
Question	49	*What are the factors in which the degree of processing of the n-linked oligosaccharides depends?*
Piece	31	In some cases the first processing reaction (removal of mannose residues) does not occur, which prevents the following addition of carbohydrate residues, leading to the formation of oligosaccharides rich in mannose instead of complex oligosaccharides that follow the full processing pathway.
Question	50	*What type of error may occur in the processing pathway of the n-linked oligosaccharides?*
Question	51	*What type of molecules are formed in the case of first reaction errors?*
**Flashcard**	**24**	
Piece	32	In the proteins destined to the lysosomes, phosphorylation of mannose residues in two sequenced reactions.
Question	52	*What is the type of reaction that occurs in the proteins destined to the lysosomes?*

**Flashcard**	**25**	
Piece	33	In the proteins destined to the lysosomes, the first reaction is catalyzed in the cis face by the enzyme n-acetylglucosamine phosphotransferase.
Question	53	*What is the first modification in the proteins destined to the lysosomes?*
Question	54	*What is the enzyme responsible for the first modification that occurs in the proteins destined to the lysosomes?*
Piece	34	The n-acetylglucosamine phosphotransferase transfers a group n-acetylglucosamine phosphate to the mannose residues of the lysosomal hydrolases.
Question	55	*That is the molecule transferred by the n-*acetylglucosamine *phosphotransferase?*
Question	56	*What is the molecule that accepts the n-acetylglucosamine phosphate transferred by the enzyme n-*acetylglucosamine *phosphotransferase?*
**Flashcard**	**26**	
Piece	35	The second reaction is catalyzed by a phosphodiesterase that removes the n-acetylglucosamine group, leaving behind a phosphorylated mannose residue.
Question	57	*What is the second modification in the proteins destined to the lysosomes?*
Question	58	*What is the enzyme responsible for the second modification that occurs in the proteins destined to the lysosomes?*
**Flashcard**	**27**	
Piece	36	Processing specificity of lysosomal proteins resides in the n-acetylglucosamine phosphotransferase enzyme, that catalyses the reaction of addition of n-acetylglucosamine phosphate.
Question	59	*What is the molecule responsible for the specificity of the lysosomal protein processing?*
Question	60	*What is the reaction catalyzed by the n-*acetylglucosamine *phosphotransferase?*
Piece	37	This enzyme recognizes a structural determinant present uniquely in the lysosomal proteins, named "signal patch", formed by the juxtaposition of amino acid sequences from different regions of the polypeptide chain, as illustrated in the picture.
Question	61	*How is the structural determinant present only in the lysosomal proteins named?*
Question	62	*What is the lysosomal protein structure recognized by the enzyme n-*acetylglucosamine *phosphotransferase?*
Question	63	*What is the composition of the structural determinant present in the lysosomal proteins?*
Image	5	Reckoning and processing of the lysosomal hydrolases by the n-acetylglucosamine phosphotransferase (GlcNAc phosphotransferase)
